# The relationships between body mass index, reciprocal ponderal index, waist-to-height ratio, and fitness in young adult males

**DOI:** 10.3389/fpsyg.2023.1250913

**Published:** 2023-10-12

**Authors:** Mehdi Ben Brahim, Alejandro Sal-de-Rellán, Ariadna Hernaiz-Sánchez, Hussain Yasin, Adrián García-Valverde

**Affiliations:** ^1^Department of Health and Physical Education, Prince Sultan University, Riyadh, Saudi Arabia; ^2^Faculty of Health Sciences, Universidad Isabel I, Burgos, Spain; ^3^Faculty of Social Sciences and Communication, Universidad Europea de Madrid, Madrid, Spain

**Keywords:** anthropometric measures, body measurements, physical test, adolescence, physical fitness, indexes

## Abstract

**Background:**

Anthropometric measures such as the body mass index (BMI), reciprocal ponderal index (RPI), and waist-to-height ratio (WHR) have been proposed as predictors of physical fitness. This study aimed to identify the differences in explanatory capacity and fit of BMI, RPI, and WHR on physical fitness, which involves jumping, sprinting, change of direction, and aerobic capacity, by adjusting the polynomial regression.

**Methods:**

A sample of 297 healthy, recreationally active male university students between 18 and 20 years old was recruited for this study. Anthropometric measurements (height: 174.09 ± 6.27 cm, weight: 78.98 ± 20.27 kg, waist circumference: 93.74 ± 14.56 cm) were taken for each participant. Jumping tests (squat jump, countermovement jump), sprinting tests (20 m sprint), agility tests (agility T-test), and aerobic/endurance tests (6 min walk test, VAM-EVAL test) were performed. Nonlinear quadratic regression models were used to assess the relationship between the jump, sprint, and fitness test scores and the anthropometric indices. The models were compared based on R-squares and Bayesian Information Criterion (BIC). The significance level was set at *p* < 0.05.

**Results:**

The results showed that all the indices predicted a portion of the variance because all variables and index relationships were significant. Regarding the fitted models, the Bayesian Information Criterion showed that BMI was the best indicator of performance, although the RPI was better for VO_2max_.

**Conclusion:**

These findings may be of great interest to practitioners because it appears that anthropometric measures can be used to predict physical fitness in certain tests although the accuracy raises any concerns.

## 1. Introduction

The identification of talented athletes is a critical component of successful athletic development programs. Previous studies have shown that early talent identification can help athletes achieve their full potential (Sarmento et al., 2018; Mendes et al., 2022). Several methods to identify physical talent have been applied, including physical measurements (e.g., motor performance tests), psychological assessments (e.g., personality and motivation inventories), and anthropometric measures (Muñoz et al., 2020; Robertson et al., 2022). Many researchers have conducted studies to identify talent in different team sports, such as futsal (Mendes et al., 2022) and soccer (Sarmento et al., 2018; Obetko et al., 2019). Also in individual sports, such as badminton (Robertson et al., 2022) and taekwondo (Wazir et al., 2019), and in elite male young runners (Muñoz et al., 2020). Other studies have demonstrated a relationship between several body shape indices and physical fitness in sports (Watts et al., 2012; Cossio-Bolaños et al., 2022; García et al., 2022; Negra et al., 2022). In this sense, anthropometric measurements such as the relationship between body weight and height, including the body mass index (BMI), reciprocal ponderal index (RPI), and waist-to-height ratio (WHR), have been proposed as predictors of physical fitness in several regression models (Lopes et al., 2017; Cossio-Bolaños et al., 2022), providing valuable information to practitioners to assess an athlete’s body shape (García et al., 2022) and identify potential risk factors for injury (Oh et al., 2023). However, the ability to predict physical fitness using these indices has not been compared.

BMI has been widely used to quantify body fat and identify people who are overweight (Qin et al., 2022) which have negative consequences for the health of children (e.g., dyslipidemia, arterial stiffness, and less cardio-respiratory capacity) (Vaara et al., 2013; Abdelkarim et al., 2020) and adults (e.g., risk of heart attack) (Jafari et al., 2019). Several studies have identified that a high BMI is related to poor cardiorespiratory fitness and vertical jump (Qin et al., 2022; Manzano-Carrasco et al., 2023), which is linked to effects by the kind of sport done on physical fitness (Tahira, 2021). In this sense, regular sport participation could be a great strategy to increase fitness and combat the consequences of high BMI (Tahira, 2021). However, because length and volume are unidimensional and tridimensional constructs, there may be an incorrect dimensional comparison related to height and weight, respectively (Ross et al., 2000). Therefore, other indexes, such as the RPI, have been proposed. The RPI is believed to adjust to an allometric model (Ricardo and De Araújo, 2002) owing to its calculation based on the cubic dimension of body weight. Although there is limited research on RPI in the context of fitness assessments, it is speculated that an optimal RPI score may vary depending on the test (e.g., jump and strength assessment); therefore, the RPI should be used with caution (Silva et al., 2016). Other indexes, such as the WHR, could be feasible (Ross et al., 2000) as this measure has shown a relationship with some fitness markers (e.g., deep squat, in-line lunge) (Lavados et al., 2020). Nevertheless, the relationship between these indices and fitness tests seems to be influenced by several factors, as it has shown a nonlinear distribution in all studies (Lopes et al., 2017; Cossio-Bolaños et al., 2022). Although the use of the aforementioned tests has been widely studied, so far, these tests have not been used as indicators of the physical condition of young athletes. This knowledge could be of interest in order to establish future lines of training based on the results obtained in these tests.

Many authors have used linear regression to build predictive models based on the BMI (Chen et al., 2020; Sun et al., 2022) and RPI (Nevill et al., 2019; Cossio-Bolaños et al., 2022). However, traditional linear regression assumes a linear relationship between variables, which may not fully capture nonlinear patterns in body shape index data (Chen et al., 2020). To address this, Lopes et al. (2017) proposed the use of polynomial regression to enhance the fitting of the equation to the data distribution and increase the predictability of the indexes. Polynomial regression can help identify the optimal value of the index that may be associated with enhanced performance or increased health risks (Krakauer and Krakauer, 2012). Therefore, polynomial regression has emerged as a valuable statistical technique that could allow a more comprehensive understanding of the complex relationship between body shape index and fitness tests, and both linear and nonlinear trends could be recorded (Hastie et al., 2009).

Accordingly, this study aimed to identify the explanatory capacity and fitting of the BMI, RPI, and WHR on physical fitness, which involves jumping, sprinting, change of direction, and aerobic capacity.

## 2. Materials and methods

### 2.1. Participants

In total, 297 healthy, recreationally active male university students between 18 and 20 years (19.15 ± 0.85) were enrolled in this study. In order to sort them into this category, they were asked if they accomplished the minimal physical activity recommended by WHO. The descriptive data of the participants are summarized in Supplementary Table S1. None of the participants reported any injury, disease, or supplement intake that could have influenced their performance. All participants signed an informed consent form that explained the potential risks of the study. The study was conducted in accordance with the Declaration of Helsinki and approved by the ethics committee of the authors’ university before recruitment.

### 2.2. Procedures

Participants visited sports facilities three times on non-consecutive days. On the first day, anthropometric measurements and jump tests were performed. Sprint and agility tests were performed on the second day, and endurance tests were performed on the third day. All the participants performed a standardized warm-up involving yoga, dynamic stretching, jumps, and progressive sprints on all the test days. All participants were verbally encouraged to perform every test as well as possible to achieve the best record. The facility where the tests were performed was kept between 20 to 25°C.

### 2.3. Anthropometry and indexes

According to the ISAK statements, height, body weight, and waist perimeter were measured for each participant (Norton, 2018). Height was measured using a stadiometer (Holtain Ltd., Crymych, UK) with an accuracy of 1 cm. Body weights were measured using an electronic scale (Seca Instrument Ltd., Hamburg, Germany). The waist perimeter was measured using an anthropometric steel tape (Cescorf, Porto Alegre, Brazil) at the end of normal expiration. After measurement, the indices were calculated, as shown in Supplementary Table S2.

### 2.4. Jumping test

Three attempts at the squat jump (SJ) and countermovement jump (CMJ) were performed with a rest period of 45 s between each attempt. An electronic contact mat system at 1000 Hz (Globus Ergo Tester, Codognè, Italy) was used to record the jumps. All participants were asked to place their hands on their hips during each test jump and keep their knees straight during landing. Correct jump performance was visually standardized at a 90° angle by using a manual protractor (Westward, China) for each participant’s knees by a researcher. To ensure accuracy, a bungee band was placed under the participants’ glutes, and the participants’ glutes were required to contact the band before jumping. The highest jump was selected for analysis.

### 2.5. Sprinting test

Participants were asked to perform a 20 m sprint twice after resting for 5 min, and the shorter sprint time was selected for analysis. Participants had to start 0.5 m before the first photocell while standing with their feet together within 1 min of the researcher’s signal. Photocells (Cell Kit Speed, Brower, USA) were placed at 0, 5, and 20 m to measure 5 m and 20 m sprints.

### 2.6. Agility test

The agility T-Test was performed according to the method described by Semenick (1990). The participants stood with their feet together 0.5 m before the single photocell (Cell Kit Speed Brower, USA), set to start/stop at 0 m. Three attempts were made; however, only the best attempt of each participant was included in the analysis.

### 2.7. Aerobic/endurance test

Two tests were used to assess the aerobic capacity. First, a 6 min walk test was performed according to the method described by Enright (2003), with the total distance walked by each participant recorded. Subsequently, the VAM-EVAL test was performed according to the method described by Cazorla and Léger (1993), and the total distance and last velocity reached were recorded. In addition, the estimated oxygen consumption was calculated using Leger and Mercier’s (1984) equation based on the velocity achieved in the VAM-EVAL test.

### 2.8. Statistical analysis

The data is presented as a plot distribution and shows the relationship between the variables. A test-specific regression analysis was performed for each index. A nonlinear quadratic model was used to assess the relationship between the jump, sprint, and fitness test and index scores, with each fitness item-dependent variable, index, and squared index being considered as independent variables (each regression equation is indicated in each figure). The models were compared based on R-squares and Bayesian Information Criterion (BIC). The BIC was interpreted as a good fit when a lower value was provided (Neath and Cavanaugh, 2012). All tests and plots were constructed using RStudio (base R and stats packages for analysis and ggplot2 package for illustrations), and the significance level was set at *p* < 0.05.

## 3. Results

**Figure 1:**
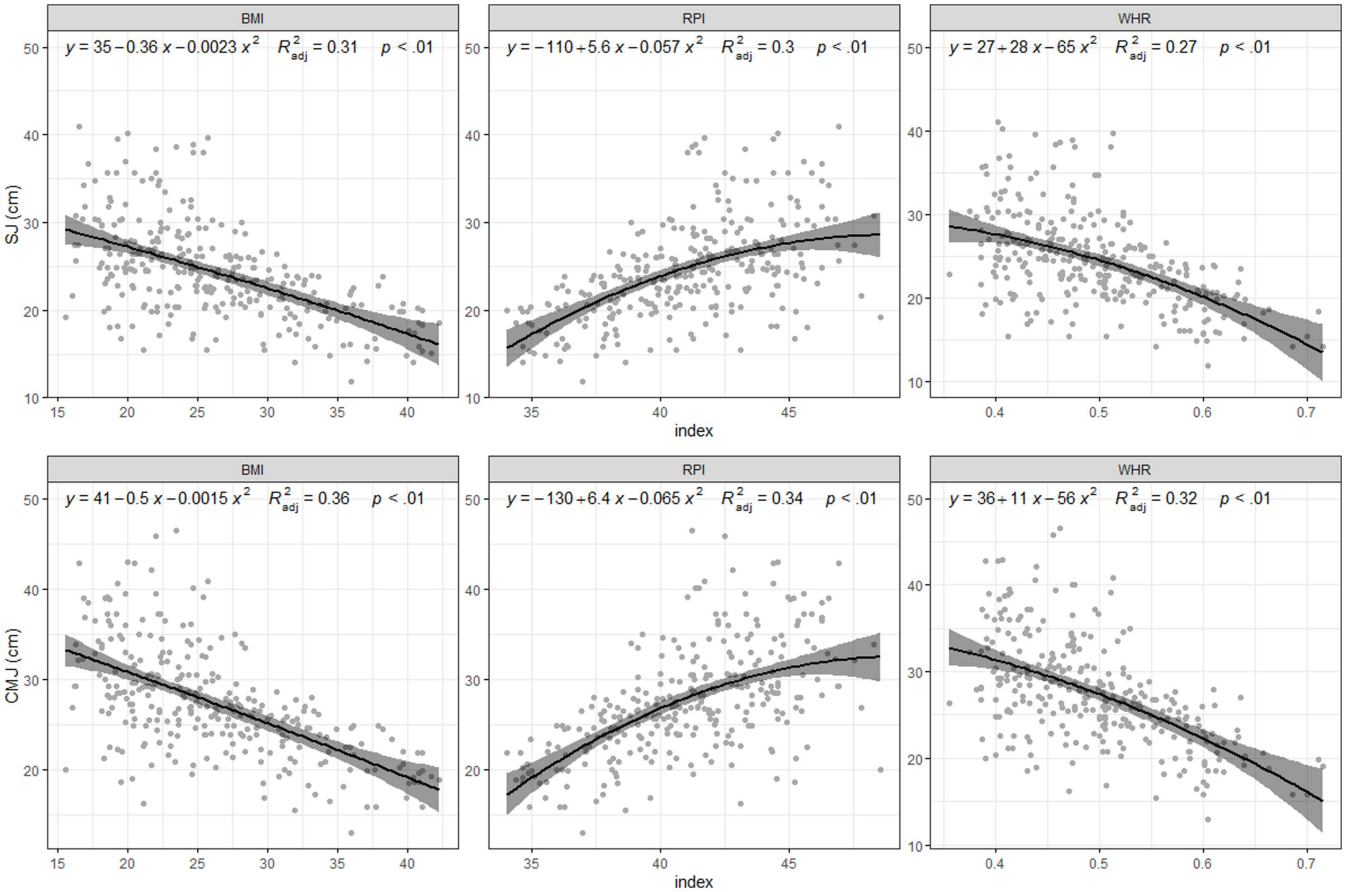
Jump.

**Figure 2:**
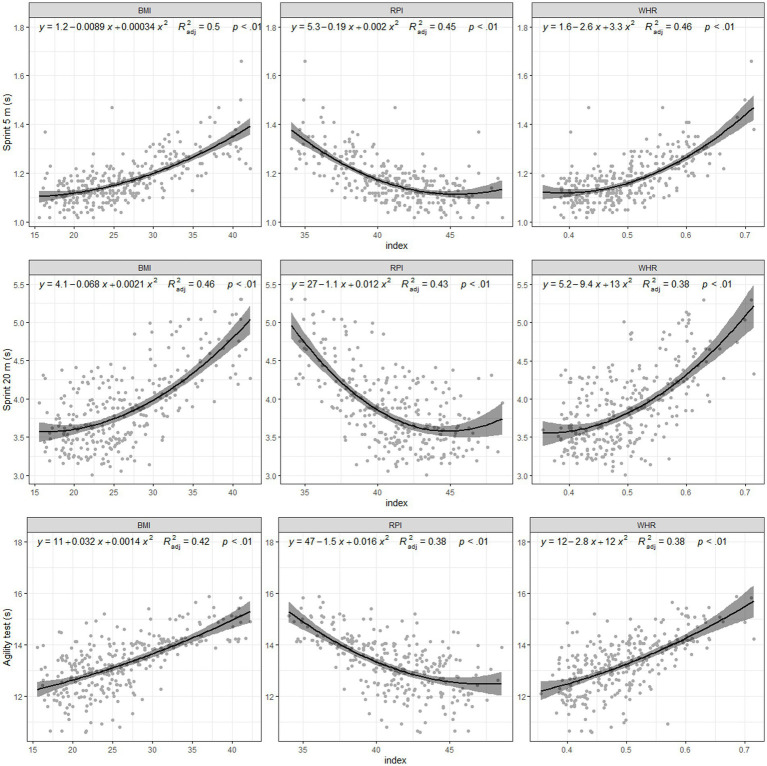
Sprint.

**Figure 3:**
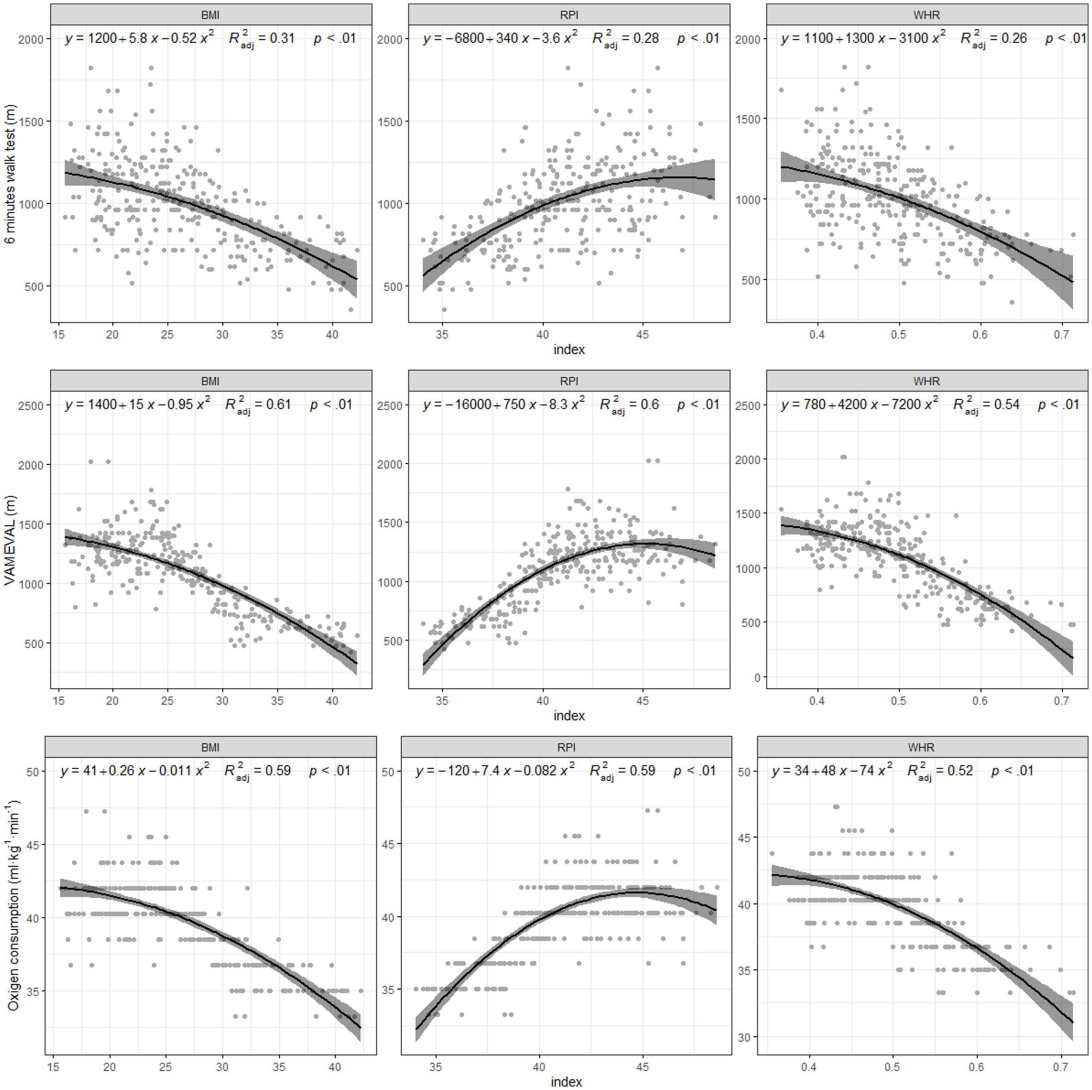
Fitness.

The results showed that each index provided a different trend in parameter prediction. In this sense, results showed a positive relationship between sprint tests and indexes such as BMI and WHR, while RPI showed a negative trend in all tests. However, for the jumping and fitness tests, BMI and WHR provided a negative relationship while RPI showed a positive trend. Despite of this fact, all indices explained a small portion of the variance, as all variables and index relationships were significant. –These indices explained nearly 30% of the variance in SJ and CMJ (Figure 1). In sprinting and agility, 38–46% of the variance was based on the indices, although BMI predicted up to 50% of the variance in 5 m sprinting (Figure 2). The variance in the 6 min walk test results could be predicted by 30% using BMI, while 54–61% of the variance in VAM-EVAL was predicted by indexes (Figure 3). Regarding the fitted models (Table 1), the BIC showed that BMI was the best predictor of performance, although the RPI was better for the VO_2max_ predicted.

**Table 1:** Bayesian information criterion (BIC) as a comparison of the model fitting so as to every index.

Measurement	BMI	RPI	WHR
SJ	**1763**	1767	1778
CMJ	**1798**	1805	1816
5-m sprint	**−751**	−723	−729
20-m sprint	**258**	276	299
Agility	**741**	757	757
6-min walk	**4,066**	4,078	4,085
VAMEVAL	**3,994**	3,999	4,041
VO_2max_	1,219	**1,216**	1,262

Bold characters indicates each measurement’s lowest value of the BIC index. SJ, squat jump; CMJ, countermovement jump.

## 4. Discussion

This study aimed to identify the explanatory capacity and fit of BMI, RPI, and WHR on physical fitness, including jumping, sprinting, change of direction, and aerobic capacity. This is the first study to compare the ability of BMI, RPI, and WHR to predict physical fitness performance in a young adult population. The most insightful finding was the different trends observed in physical fitness among indexes. In common with previous literature, our results showed that high BMI values are linked to poor performance (Nikolaidis, 2013). However, RPI showed an inverse relationship which could be explained by the meaning of body shape index. In this sense, BMI consists of identifying how many units of the subject’s square height are represented in the subject’s weight, whereas RPI consists of knowing how units of the body weight as a single dimension measure (cubic root) is represented in the subject’s height. Then body shape with high BMI values may be identified as lower RPI values. On the other hand, WHR kept a similar trend to BMI since its interpretation of body mass distribution is also elaborated based on the subject’s height. Moreover, BMI was found to influence the SJ, CMJ, 5 m sprint, 20 m sprint, agility, 6 min walk, and VAM-EVAL results. However, with regard to VO_2max,_ the best results were observed for RPI.

Regarding BMI, our findings showed superior results in SJ and CMJ than in RPI and WHR. Previous studies have shown that BMI is a good predictor of vertical jump performance, indicating that a high BMI decreases the vertical jump height (Qin et al., 2022; Manzano-Carrasco et al., 2023). Although there is still insufficient research on the relationship between jump performance and RPI, previous studies have generally reported a better fit when using RPI than BMI or WHR (Silva et al., 2016). However, our results did not support this hypothesis. This could be due to differences in the jump test applied (i.e., SJ and CMJ vs. standing long jump), which presents a different force application (i.e., vertical vs. horizontal vector) (Silva et al., 2016).

In contrast, our results showed that BMI was the best predictor of 5 m sprint, 20 m sprint, and agility performance. Previous studies have indicated that BMI is a significant predictor of sprint performance (Campa et al., 2019; Qin et al., 2022), with an inverted U-curve indicating the best performance in these tests in the middle, implying normal BMI values. However, other studies have also identified the RPI as a good predictor in the agility test, suggesting that an adequate height/body mass ratio is related to higher performance in this test (Silva et al., 2016). The best predictor for COD also appears to be BMI. Thus, our results are consistent with those of previous studies (Campa et al., 2019; Chen et al., 2020; Qin et al., 2022). However, discrepancies remain between studies, as some have identified the RPI as the best predictor of COD (Silva et al., 2016). The RPI is associated with better performance in ectomorphs, both in adolescents (Silva et al., 2016) and sprint athletes (Watts et al., 2012). However, the RPI was also associated with worse performance among ectomorph tennis players (Gale-Watts and Nevill, 2016). These differences may be attributed to the different characteristics of each sport.

In terms of the 6 min walk and VAM-EVAL tests, the best predictor was BMI. These findings are in line with previous results (Manzano-Carrasco et al., 2023). Considering the importance of cardiorespiratory fitness levels for health in the young adults males (Ortega et al., 2018; Agostinis-Sobrinho et al., 2022), BMI might be considered as a predictor of aerobic fitness tests. Furthermore, it has previously been shown that RPI also predict aerobic test performance (Thomis et al., 2000; Silva et al., 2016). Nevertheless, the results may depend on the type of test performed (Nobari et al., 2023),so both BMI and RPI may be good predictors of aerobic capacity. Furthermore, considering RPI as a predictor, the best results have been reported in taller adolescents and those with a more linear physique (Nevill et al., 2009; Nhantumbo et al., 2012). Finally, the best predictor of VO_2max_ in the current study was the RPI (Sagat et al., 2023), which is in agreement with previous studies (Thomis et al., 2000; Silva et al., 2016). These findings show that the RPI is a good predictor of aerobic capacity. There is still much disagreement regarding the best predictor of performance, and further studies should be conducted before definitive conclusions can be drawn.

This study has several limitations: (i) Oxygen consumption was estimated using Leger and Mercier’s (1984) equation based on the velocity achieved in the VAM-EVAL test; however, other tests could be more accurate. (ii) Body fat has been identified as a moderator of performance (Esco et al., 2018) thus future studies may include this parameter in the prediction equation. (iii) In addition, the participants were all from a single country; therefore, the results may not apply to participants in other countries or from other ethnic groups. (iv) The sample consisted only of male university students. Future studies should consider including female university students in the sample.

In conclusion, our results indicate that BMI is the best indicator of performance in the SJ, CMJ, 5 m sprint, 20 m sprint, agility, 6 min walk, and VAM-EVAL tests. However, the best indicator of VO_2max_ performance was the RPI. Nevertheless, the ability to the prediction of indexes is hardly 40% of the variance in many cases.

In terms of practical application, these results should be taken into account when selecting which anthropometric measures should be used to identify physical fitness in young adult males. For this reason, BMI is the best fit for vertical jump, sprint, agility and aerobic capacity tests, while RPI is the best fit for VO2max.

## Data availability statement

The raw data supporting the conclusions of this article will be made available by the authors, without undue reservation.

## Ethics statement

The studies involving humans were approved by Approval number FUi1-PI002. Universidad Isabel I, Burgos, Spain. The patients/participants provided their written informed consent to participate in this study. The studies were conducted in accordance with the local legislation and institutional requirements. The participants provided their written informed consent to participate in this study.

## Author contributions

MB, HY, AG-V, AS-R, and AH-S conceptualized and visualized the study. MB and HY performed the data curation. MB, HY, and AG-V performed the formal analysis. AS-R and AG-V designed the study. AS-R supervised the study. AS-R, AG-V, and AH-S wrote and reviewed the manuscript. All authors contributed to the article and approved the submitted version.
