# Supplementary material for: The relationships between body mass index, reciprocal ponderal index, waist-to-height ratio, and fitness in young adult males
Source: Front Psychol. 2023 Oct 12;14:1250913. doi: 10.3389/fpsyg.2023.1250913 (PMC10600486; doi:10.3389/fpsyg.2023.1250913)
Supplement: Supplementary file 1 [file Data_Sheet_1.docx]

Supplementary Tables

**Supplementary Table 1**

Descriptive of participants

|  | Mean ± SD |
| --- | --- |
| Age (years) | 19.15 ± 0.85 |
| Weight (kg) | 78.98 ± 20.27 |
| Height (cm) | 174.09 ± 6.27 |
| Waist perimeter (cm) | 93.74 ± 14.56 |
| BMI | 25.99 ± 6.24 |
| RPI | 41.10 ± 3.17 |
| WHR | 0.49 ± 0.07 |

**Supplementary Table 2**

Equations used to calculate indexes

| Index | Equation |
| --- | --- |
| Body mass index (BMI) | BW·(H/100)^-2^ |
| Reciprocal Ponderal Index (RPI) | H·BW^-1/3^ |
| Waist-to-Height ratio (WHR) | W·H^-1^ |

BW = Bodyweight (kg), H = Height (cm); W = Waist (cm)
